# Structural and biochemical insight into mode of action and subsite specificity of a chitosan degrading enzyme from *Bacillus spec*. MN

**DOI:** 10.1038/s41598-018-36213-6

**Published:** 2019-02-04

**Authors:** Ratna Singh, Tobias Weikert, Sven Basa, Bruno M. Moerschbacher

**Affiliations:** Institute for Biology and Biotechnology of Plants, University of Münster, Schlossplatz 8, 48143 Münster, Germany

## Abstract

Chitosans, partially de-N-acetylated derivatives of chitin, are multifunctional biopolymers. In nature, biological activities of partially acetylated chitosan polymers are mediated in part by their oligomeric breakdown products, which are generated *in situ* by the action of chitosanolytic enzymes. Understanding chitosanolytic enzymes, therefore, can lead to the production of chitosan oligomers with fully defined structures that may confer specific bioactivities. To address whether defined oligomer products can be produced via chitosanolytic enzymes, we here characterized a GH8 family chitosanase from *Bacillus spec*. MN, determining its mode of action and product profiles. We found that the enzyme has higher activity towards polymers with lower degree of acetylation. Oligomeric products were dominated by GlcN_3_, GlcN_3_GlcNAc_1_, and GlcN_4_GlcNAc_1_. The product distribution from oligomers were GlcN_3_ > GlcN_2_. Modeling and simulations show that the binding site comprises subsites ranging from (−3) to (+3), and a putative (+4) subsite, with defined preferences for GlcN or GlcNAc at each subsite. Flexible loops at the binding site facilitate enzyme-substrate interactions and form a cleft at the active site which can open and close. The detailed insight gained here will help to engineer enzyme variants to produce tailored chitosan oligomers with defined structures that can then be used to probe their specific biological activities.

## Introduction

Chitosans, a family of partially de-*N*-acetylated derivatives of chitin, are among the most versatile and most promising functional biopolymers, with interesting physicochemical properties and multiple bioactivities such as plant disease protectants^1,2^, antimicrobial agents^3^, as sustained release nanoparticle carriers for drug, gene, or vaccine delivery^4^. As chitosans are linear co-polymers of β-1, 4-linked *N*-acetyl-D-glucosamine (GlcNAc) and D- glucosamine (GlcN) units, they have differing degrees of polymerization (DP), degrees of acetylation (DA), and patterns of acetylation (PA). However, these parameters crucially determine both the physico-chemical properties of the partially acetylated chitosan polymers as well as the biological activities of the polymers and their oligomeric breakdown products^1,5^. While polymers’ and oligomers’ DP and DA can be controlled to some extent, commercially available chitosan polymers invariably have random PA^6,7^. Hence, while the influence of DP and DA on chitosan’s biological activities is currently studied intensely, the influence of PA is completely unknown. Therefore, chitosan research has recently focused on producing oligomers with at least a partially known and defined PA through using chitin- and chitosan-modifying enzymes, such as sequence-specific chitosan hydrolases or regio-selective chitin deacetylases^1,8^, these enzymes can be a tool to understand the influence of PA on bioactivities.

Of these enzymes, chitosan hydrolases can be divided into chitinases, chitosanases and, possibly, chitinosanase^9^. Depending on their subsite specificities, the resulting oligomeric products will have defined GlcN or GlcNAc units at their reducing and/or non-reducing ends. All chitinases are able to cleave the glycosidic linkage between two acetylated GlcNAc units but not the linkage between two GlcN units, while all chitosanases are able to cleave between two non-acetylated GlcN units but not between two GlcNAc units, with possible exceptions^10^. Although chitinases have been extensively studied^11,12^ much less is known on the more diverse chitosanases, which are produced by a variety of bacteria and fungi^13^ and by viruses^14^. In the past, chitosanases have long been classified into four classes based on their GlcN/GlcNAc specificities^15^. While all chitosanases hydrolyze the linkage between two GlcN units^16^, class I chitosanases also cleave the GlcNAc-GlcN bond (cleavage site X│D, with X = GlcN or GlcNAc; D = GlcN), and class III enzymes also cleave the GlcN-GlcNAc bond (D│X). Class II chitosanases are thought to only cleave the GlcN-GlcN bond (D│D), while class IV enzymes cleave all linkages except for the GlcNAc-GlcNAc bond. However, a recent study revealed that chitosanase specificities are much more diverse than this classification suggests^17^. In particular, chitosanases also differ in their specificities or preferences for GlcN or GlcNAc subunits at subsites (−2) and (+2). Apart from the above classification groups based on cleavage site, chitosanases have also been grouped based on sequence similarity, resulting in six glycoside hydrolase (GH) families, namely GH5, GH7, GH8, GH46, GH75, and GH80; chitosanases of GH46 have been studied most extensively^12,18^. So far, no direct relationship has been established between the above-mentioned chitosanase classes I-IV and the GH families. This broad sequence diversity in the chitosanase family, which greatly exceeds that of chitinases, raises the prospect of studying their subsite specificities in the hope of finding more specific enzymes that might be useful for producing chitosan oligomers with more defined PA.

To further this goal, in this study we focused on a bacterial chitosanase from *Bacillus spec*. MN (CSN-MN)^19^ belonging to the GH8 family. In the present study, we aim to investigate the structural and molecular features of CSN-MN that control its activity, specificity, and relative mode of action. To this end, recombinant CSN-MN enzyme was purified and incubated with well-defined chitosan polymers as substrates. Subsequently, the oligomeric products were analyzed using thin layer chromatography and mass spectrometry In parallel, molecular dynamic simulation combined with ensemble docking was carried out and possible subsites, the property of the substrate binding site, functional residues, their interactions with the substrate, and subsite vs. subunit specificities were defined. Furthermore, principle component analysis (PCA) was carried out to study the functional motion of loops at the active center in substrate-free and substrate-bound CSN-MN. Merging data obtained from *in silico* and *in vitro* experiments provided detailed insights into CSN-MN’s subsite specificities and mode of action; these insights will guide future enzyme engineering^20^, to produce well-defined chitosan oligomers that can be used to understand structure-function relationships of partially acetylated chitosan oligomers.

## Results and Discussion

### Hydrolysis of fully deacetylated chitosan oligomers (GlcN_n_)

**Figure 1:**
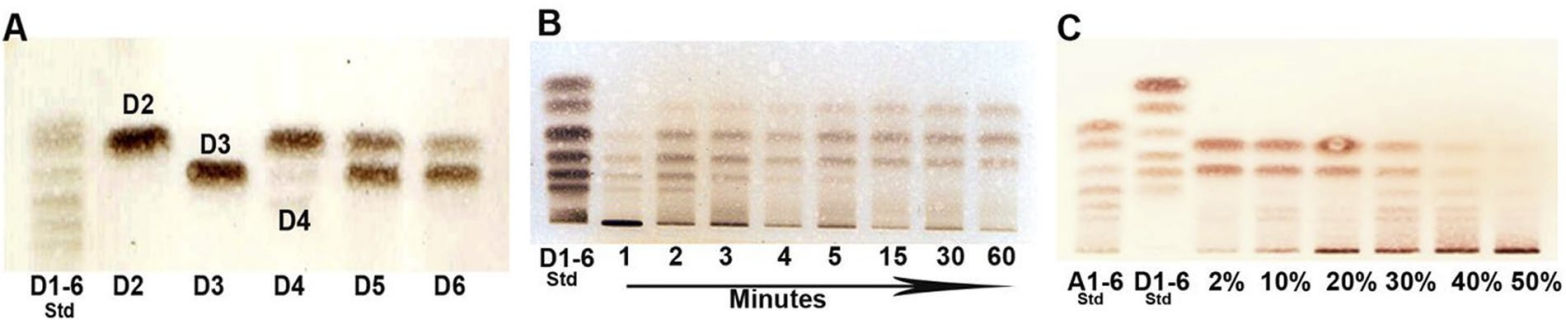
Chitosan oligomer and polymer degradation with CSN-MN. GlcNAc (A1–6) and GlcN (D1–6) oligomers were used as standards (Std). For clarity of bands, brightness and controls were adjusted and equally applied across the entire image. Uncropped images at multiple exposures are presented in supplementary Fig. S1. (**A**) Thin layer chromatography of chitosan oligomers obtained by digestion of GlcN oligomers with CSN-MN revealed the degradation of only tetramer, pentamer, and hexamer. (**B**) Degradation of chitosan polymers of DA 0% at different time points of incubation with CSN-MN shows gradual occurrence of first larger than smaller oligomers, indicating endo-mode of enzyme action. (**C**) A range of chitosan polymers with varying DA from 2 to 50% affirmed a decrease in hydrolysis with increasing DA.

Enzymatic hydrolysis of oligomer assessed by thin layer chromatography (TLC) showed that neither the dimer (GlcN_2_) nor the trimer (GlcN_3_) were cleaved by CSN-MN, while the tetramer (GlcN_4_) was almost exclusively cleaved into dimers, and the pentamer (GlcN_5_) into a dimer and trimer (Fig. 1A). For the hexamer GlcN_6_, band intensities indicated that its hydrolysis predominantly led to the release of trimers and, to a lesser extent, dimers and tetramers, the latter being hydrolyzed further into dimers. The survival of some GlcN_4_ substrate at the end of the reaction indicated that GlcN_4_ was not a preferred substrate for the enzyme; conversely, GlcN_5_ and GlcN_6_ were completely cleaved into products. Time course studies using a fully deacetylated chitosan polymer (polyglucosamine, DA 0%) as a substrate showed that larger oligomers appeared gradually at first, then smaller oligomers appeared, revealing that the enzyme acts in a non-processive endo-mode (Fig. 1B).

### Enzyme kinetics

**Figure 2:**
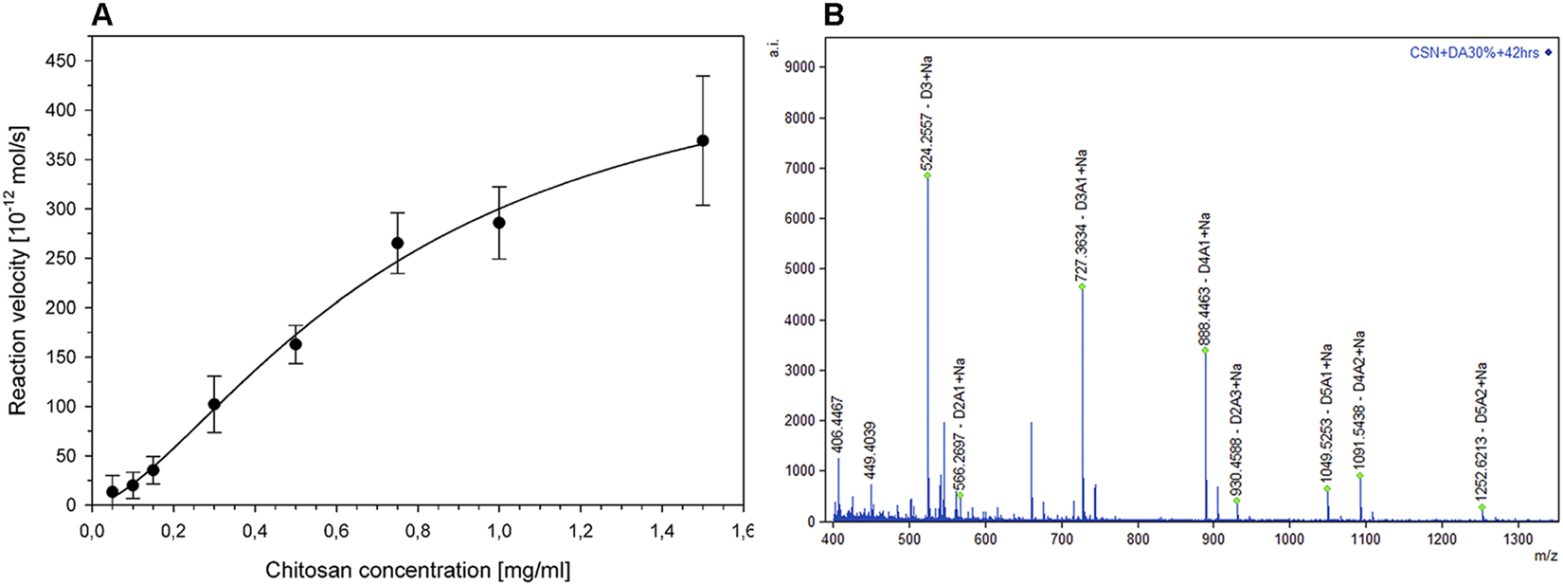
Enzyme kinetics and MS spectrum for product pattern. (**A**) Kinetics of CSN-MN-catalyzed cleavage of a chitosan polymer with a DA of 10%. Kinetics data showed a sigmoidal curve of the Hill reaction, where K_M_ was 0.74 mg/ml and kcat was 163 s^−1^, with a Hill coefficient of 1.54. Data are means of twelve (*n* = 12) independent experiments ± s.d. (**B**) Mass spectrum of oligomeric products obtained by CSN-MN-catalyzed hydrolysis of a chitosan polymer with a DA of 30%; D, GlcN; A, GlcNAc. The major products identified after hydrolysis were GlcN_3_, GlcN_2_GlcNAc_1_, GlcN_3_GlcNAc_1_, and GlcN_4_GlcNAc_1_ (see also Fig. S3). Data shown are representative of results obtained in three independent experiments.

Enzyme kinetics of CSN-MN were determined using a polymeric substrate with a DA of 10%. The kinetics showed a sigmoidal curve (Fig. 2A), where K_M_ was determined as 0.74 mg/ml and k_cat_ as 163 s^−1^, with a Hill coefficient of 1.54. As the enzyme does not appear to form multimers (Fig. S2), this could be the result of conformational changes that occur during enzyme-substrate interaction, such as loop reorganizations^21,22^ which have been observed for a chitosanase from *Pseudomonas sp*.^23^.

### Hydrolysis of partially acetylated chitosan polymers

A series of chitosan polymers with increasing DA ranging from 2% to 50% was incubated with CSN-MN. TLC of the products revealed a shift with increasing DA of the substrate, from almost exclusively dimers and trimers to oligomers with DP > 10, which were not resolved by TLC (Fig. 1C). The structural properties of the resulting chitosan oligomers in terms of their DP and DA were analyzed using MALDI-TOF MS. When DA 30% chitosan was used as a substrate, the main products identified were GlcN_3_, GlcN_2_GlcNAc_1_, GlcN_3_GlcNAc_1_, GlcN_4_GlcNAc_1_, GlcN_5_GlcNAc_1_ and GlcN_4_GlcNAc_2_ (Fig. 2B). Remarkably, the major hydrolysis products identified when chitosan polymers with DA 2%, DA 10%, DA 20%, and DA 40% were used as substrates, were also GlcN_3_, GlcN_3_GlcNAc_1_, and GlcN_4_GlcNAc_1_ **(**Fig. S3). These results, in particular the almost complete absence of double-acetylated products when a chitosan of DA 40% was used as a substrate, seem to indicate that the enzyme accepts acetylated GlcNAc groups only at very restricted subsites.

**Figure 3:**
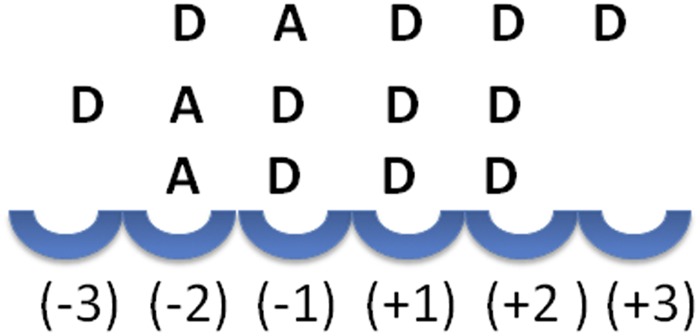
Subsite specificity from MS^2^. Accumulation of the products ADDD (76%) and DADDD (85%) disclosed that these oligomers were not used as substrates by the enzyme, revealing a strong specificity for GlcN at subsites (−1) and (−2).

To further investigate the product pattern, we next sequenced the two abundant partially acetylated products generated from enzymatic cleavage of the DA 30% chitosan polymer, i.e. the mono-acetylated tetra- and pentamers GlcN_3_GlcNAc_1_ and GlcN_4_GlcNAc_1_ (see Fig. 2B), using MS^2^ (Table 1). As expected, the reducing ends of all oligomers invariably consisted of GlcN units, indicating an absolute specificity for GlcN at subsite (−1). Furthermore, regarding the (−2) subsite, the accumulation of products ADDD (76%) and DADDD (85%) indicated that in addition to the (−1) subsite, the (−2) subsite also has strong preference for GlcN (Fig. 3). At the non-reducing ends, most oligomers had deacetylated GlcN units but some were detected with acetylated GlcNAc units, indicating that the (+1) subsite of CSN-MN is not absolutely specific for GlcN. Additionally, the linear decrease of cleavage with increasing substrate DA (Fig. S4) as well as the relative abundance of GlcN units over GlcNAc units at the products’ non-reducing ends show that at the (+1) subsite, the enzyme tends to strongly prefer GlcN. Indeed, when samples were taken early during incubation (15 min), only fully deacetylated products were detected, showing a preference of GlcN at all subsites. Subsequently, to illuminate the corresponding specificity of CSN-MN, we applied a bioinformatics approach to explore the mode of action at the structural level.

**Table 1:** MS and MS^2^ analysis of products obtained from enzymatic cleavage of DA 30% chitosan polymer.

Oligomers	MS results	MS-MS results
tetramer	GlcN_3_GlcNAc_1_ (D3A1)	76% ADDD 29% DADD 5% DDAD
pentamer	GlcN_4_GlcNAc_1_ (D4A1)	85% DADDD 15% DDADD

### 3D Model of the CSN-MN Enzyme

**Figure 4:**
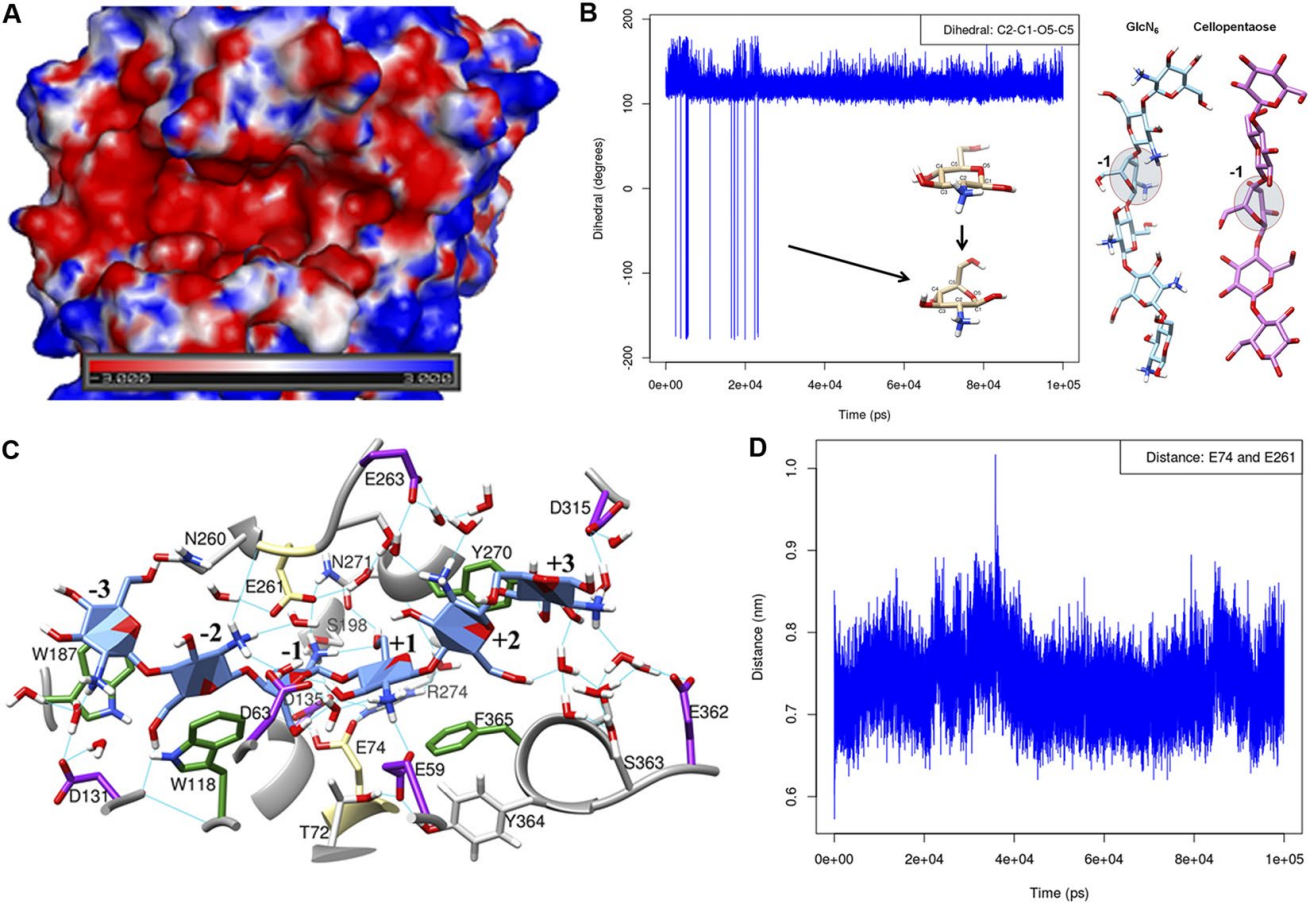
Molecular modeling studies defining the property of the binding site. (**A**) Electrostatic surface potential map of CSN-MN representing the very acidic binding site; red, acidic regions; blue, basic regions. (**B**) The 4C1 chair conformation was observed as the major conformation during simulation, changes in the dihedral angles at the different time points of simulation at subsite (−1) indicate switches in the pyranose ring conformation (see also Fig. S5). (**C**) Enzyme-substrate interaction map between CSN-MN and GlcN_6_ as a substrate showed all possible interactions; catalytic residues E74 and E261 are highlighted in khaki color, aromatic residues making stacking interactions with the substrate are colored in green, and acidic residues are colored in violet. (**D**) The distance measured between the catalytic residues E74 and E261 during simulation (average 7.4 Å) indicates that CSN-MN is an inverting enzyme.

The 3D structure of CSN-MN was modeled based on the crystal structure of the enzyme *Bsp*K17^24^. The calculated RMSD value between target and template structures was below 0.25 Å, thus the model generated showed good agreement between the two enzymes in secondary structure elements. Comparing the CSN-MN model with the *Bsp*K17 template, we identified that despite the 96% sequence identity between the two enzymes, there are conspicuous differences at the substrate-binding site. CSN-MN carries asparagine (N) and serine (S) at positions 260 and 305 in the active site, respectively, whereas the corresponding positions in *Bsp*K17 are occupied by aspartate (D) and arginine (R). As these residues are quite different in nature, we assume that these substitutions could influence subsite affinities, in particular at subsites (−2) and (+3), and, thus, product patterns may differ even between these two very closely related enzymes. Nevertheless, the active site (Fig. 4A) of CSN-MN is a wide and open cleft, which is typical for endo-acting enzymes. The three-dimensional electrostatic surface potential map shows a highly acidic active site (Fig. 4A), providing a reason for the preferential binding of highly deacetylated stretches of substrate.

### Enzyme substrate interactions map from distorted pyranose ring

To investigate the induced fit mechanism of the enzyme, molecular dynamics simulations (MD)^25^ were performed on substrate-free and substrate-bound enzymes. Prior to simulation, the enzyme-substrate complex was generated with docking, taking GlcN_6_ as the substrate. The correct pose/orientation of the GlcN_6_ oligomer at the binding site was predicted based on the lowest docking score as well as by comparing the obtained docked conformation with the crystallographic structure of GH8 endoglucanase CelA (Fig. S5) bound with cellopentaose (as the closest structural homolog to CSN-MN available, with 30% sequence identity)^26^. Further, molecular dynamic (MD)^25^ simulations were carried out over a period of 100 ns each on both substrate-free and -bound CSN-MN. After simulation, convergence to equilibrium was assessed by the root mean square deviation (RMSD), the radius of gyration and the potential energy. As overall RMSD measured from Cα throughout the simulation remained below 2 Å (Fig. S6A), and the radius of gyration (Fig. S6B) and potential energy also remained stable (Fig. S6C) in all the three independent simulations, this clearly indicated the stability and the convergence of the trajectories.

**Figure 5:**
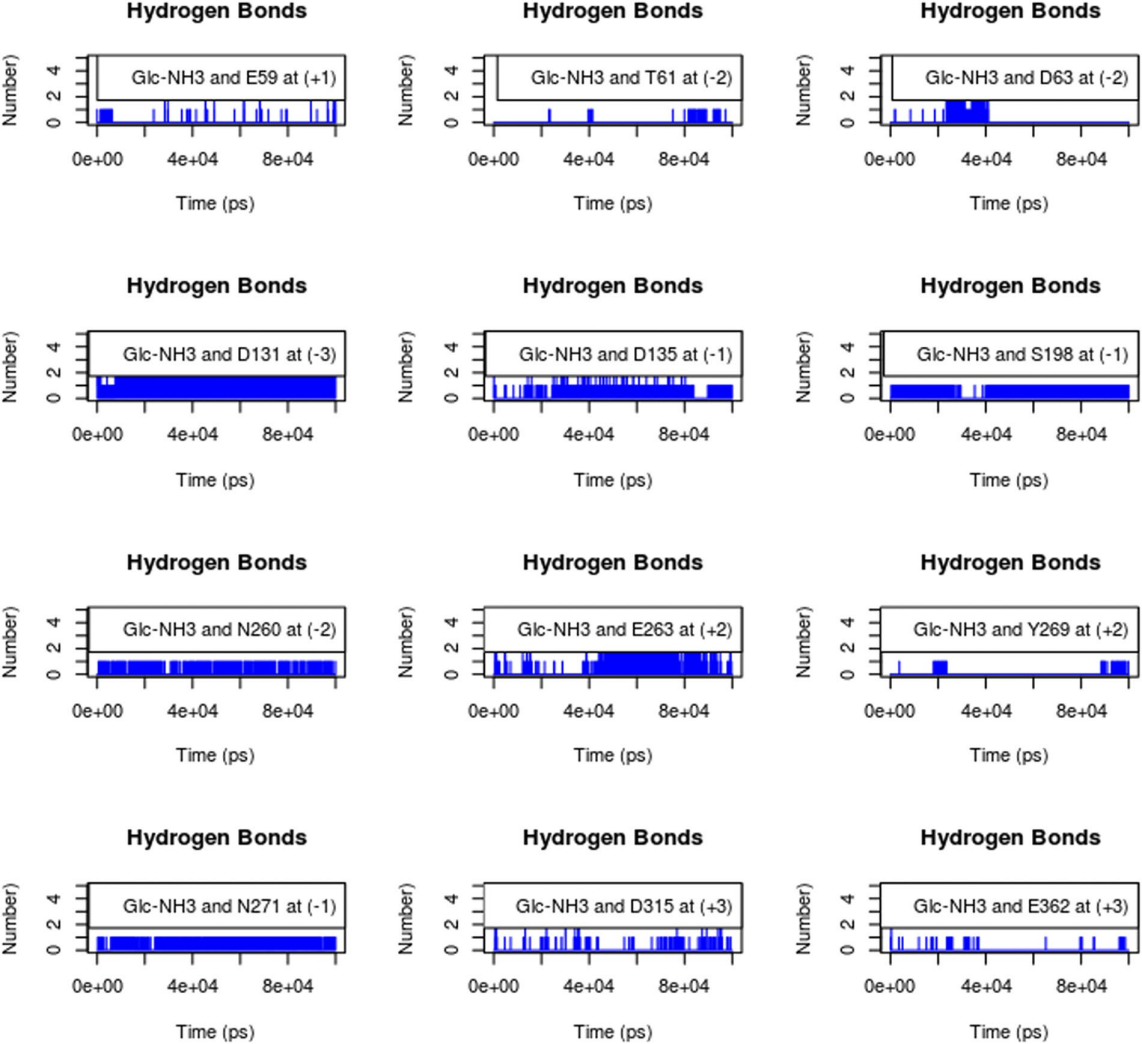
Stable hydrogen bonds between enzyme and substrate during simulation. Time series of hydrogen bonds calculated between the protonated amine of substrate subunits and subsite amino acid residues. Residue E59 present at subsite (+1), D131 at subsite (−3), D135 at subsite (−1), N260 at subsite (−2), and N271 at subsite (+1) subsite showed the most stable H-bonds throughout the simulation.

Reaction mechanisms of glycoside hydrolase (GHs) enzymes are well established. It is known that in several GHs before hydrolysis, the substrate’s carbohydrate unit bound at subsite (−1) adopts energetically higher conformations than the relaxed ^4^C_1_ chair conformation of a sugar ring. In the transition state, the energetically higher distorted ring orients the glycosidic linkage towards the catalytic acid/base residue, thus facilitating a nucleophilic attack on the anomeric carbon^27^. To identify the ring distortion during the enzyme-substrate simulation, we calculated the dihedral angles among the C2–C1–O5–C5 atoms of the (−1) sugar subunit, where a change in the dihedral angles signifies the switch in the pyranose ring conformation^28^. As expected, the results (Fig. 4B) indicated that the ^4^C_1_ chair conformation was the major conformation, while at different time points, we identified a switch from the ^4^C_1_ conformation to the distorted pyranose conformation in the (−1) subunit. The distorted conformation observed in the simulation resembles the transition state conformation of the (−1) subunit of cellopentaose (Fig. 4B) in the GH8 endoglucanase CelA structure (1KWF.pdb). The enzyme-substrate contact map generated from the distorted conformation is shown in Fig. 4C (PDB: S10). The interaction map indicates that both electrostatic and hydrophobic interactions dominate at the binding site. Residues W187, W118, F365, and Y270, present at subsites (−3), (−2), (+1), and (+2), respectively, are involved in hydrophobic interactions with the substrate. Because of their aromatic stacking interactions with the sugar rings, these residues are responsible for positioning and holding the sugar ring in the cavity (Fig. 4C). Acidic residues D131 at the (−3) subsite, D63 at (−2), D135 at (−1), E59 at (+1), E263 at (+2), and D315 as well as E362 at (+3) are involved in hydrogen bond interactions with the substrate either directly or mediated via solvent-water molecules. Additionally, S198 at (−1), N260 at (−2), Y270 at (+2), N271 at (−1), and S363 at (+3) are also involved in H-bond interaction with the substrate. Further, to get more insight into the stable hydrogen bonds during simulation, H-bonds were monitored between the protonated GlcN and the amino acid residue present at the respective subsite of the enzyme. Results suggest that amino acid residues D131 at (−3), D135 at (−1), S198 at (−1), N260 at (−2), and N271 at (−1) (Fig. 5) made the most stable H-bond with the protonated GlcN subunit. This suggests that at subsites (−3) to (−1), the enzyme prefers GlcN and these subsites contribute more to substrate binding.

Glycoside hydrolases are classified into two groups, inverting and retaining, depending on whether or not they change the anomeric conformation of the substrate upon cleavage^29^. Inverting glycosidases follow a single-step hydrolysis mechanism where the catalytic residues typically are separated by a distance of 6–12 Å^30^, while retaining glycosidases follow a two-step hydrolysis mechanism with a distance between catalytic residues of below 5.5 Å^31^. Accordingly, to assess the reaction mechanism of CSN-MN, we monitored the distance between the catalytic residues E74 and E261 in the presence of substrate. The average distance measured during simulation was 7.4 Å (Fig. 4D), indicating that CSN-MN most likely is an inverting enzyme.

### Ensemble docking and subsite vs subunit specificity

**Figure 6:**
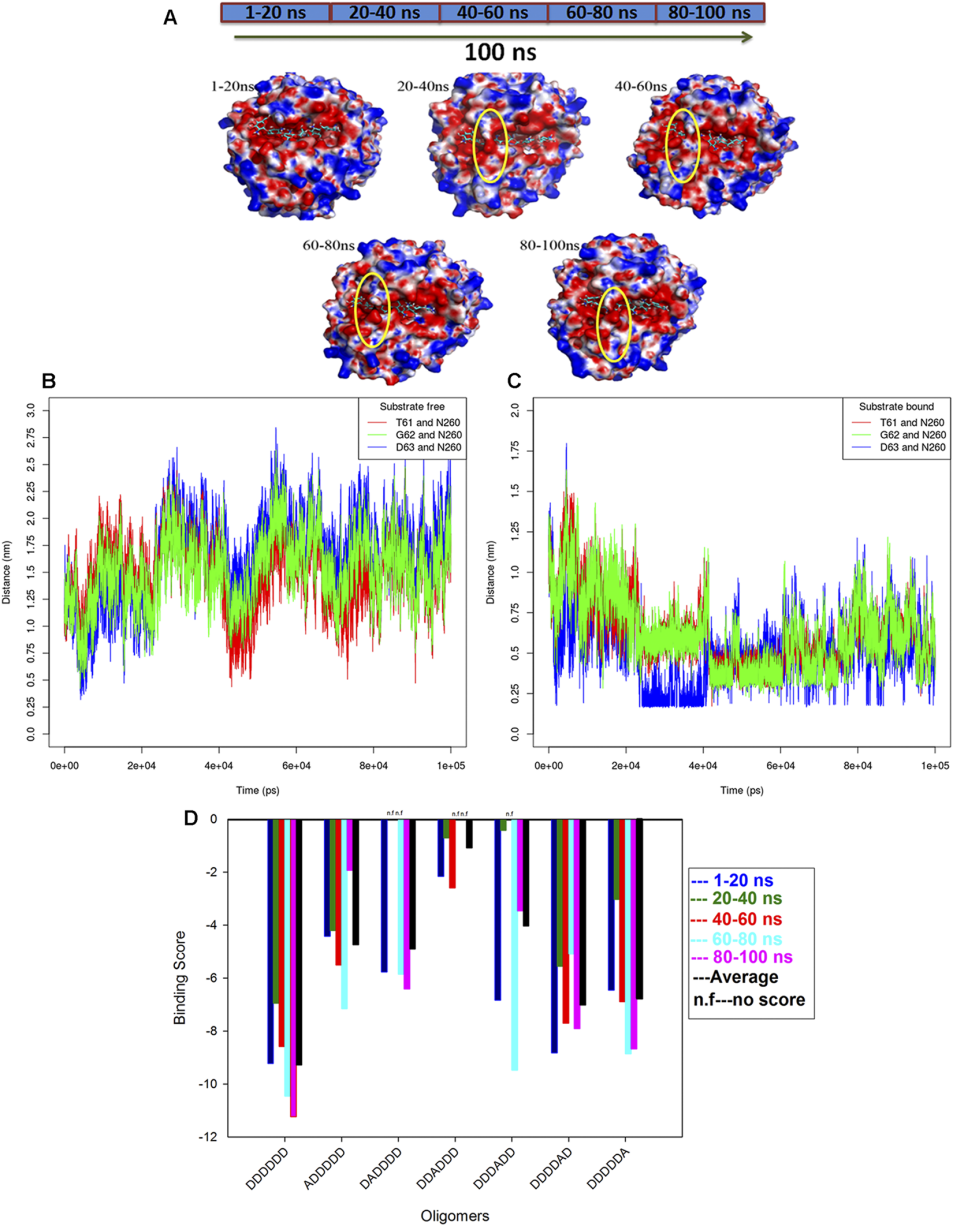
Conformational changes occur at the binding site of the enzyme in the presence of substrate, and ensemble docking results. (**A**) Average structures of substrate-bound CSN-MN derived from clusters 1–20 ns, 20–40 ns, 40–60 ns, 60–80 ns, and 80–100 ns, displaying open and closed surfaces at the binding site of the enzyme in the presence of substrate. (**B**) Distance between the amino acid residues from loop L1 (T61, G62, D63) and loop L2 (N260) in substrate-free CSN-MN, showing that during simulation, residues from loops occasionally approached closely. (**C**) Distance between the amino acid residues from loop L1 (T61, G62, D63) and loop L2 (N260) in substrate-bound CSN-MN, showing that during simulation, residues from loops approached to come into the range of interaction and were involved in forming the closed surface at subsite (−2). (**D**) Binding score of fully deacetylated chitosan hexamer to CSN-MN, displaying a change in the score with a change in the conformation of enzyme or substrate, and calculated average scores. The highest score was measured for oligomer DDDDDD, and the lowest for DDADDD, indicating subsite (−1) specificity (see also Fig. S8).

To gain a detailed understanding of subsite vs. subunit (GlcN/GlcNAc) affinity, docking studies were carried out with the fully deacetylated hexamer (DDDDD) and all six mono-acetylated hexamer substrates (ADDDD, DADDDD, DDADDD, DDDADD, DDDDAD, and DDDDDA). However, as enzyme-substrate binding follows an induced fit mechanism, whereas docking is a semi-rigid method, the effect of conformational changes occurring in the protein during substrate binding cannot be measured by simple docking simulation. Hence, here we used molecular dynamics simulations combined with ensemble docking^32,33^ for handling enzyme-substrate flexibility^34^ and to determine its effect on substrate binding. For this approach, we generated multiple conformers of the enzyme-substrate complex from the different time points of the MD simulation, and independent docking simulations were carried out on each structure with deacetylated and mono-acetylated hexamers (Fig. S7). To follow this method, the 100 ns simulation trajectory was divided into 5 parts, i.e. 1–20 ns, 20–40 ns, 40–60 ns, 60–80 ns, and 80–100 ns and then, the average substrate-bound enzyme structure was generated from each part by applying a clustering approach with cutoff 1 Å. Interestingly, the calculated structures generated from different time points of the simulation confirmed the conformational change (open and closed state) at subsite (−2) of the enzyme (Fig. 6A). Structural analyses of the enzyme-substrate complexes indicated the involvement of two loops in building a closed roof-like surface at subsite (−2). The first loop (L1) ranges from amino acid position 59 to 70 and is positioned between subsites (−2) and (+1); loop 2 (L2) ranges from amino acid 255 to 263 and is positioned between subsites (−2) and (−1). To confirm which residues from loops L1 and L2 participate in closing the cleft, we calculated the distance between the amino acid residues of L1 and L2 from the simulation trajectories. For substrate-bound CSN-MN, calculations clearly indicated that the distance between N270 from loop L2 and residues T61, G62, and D63 from loop L1 decreased and reached less than 4 Å at different time points (Fig. 6B), representing close interaction between L1 and L2 residues at subsite (−2). Conversely, for substrate-free CSN-MN, the loops remained separated and only occasionally became close to one another (Fig. 6C). Hence, we conclude that the closed surface at subsite (−2) is a substrate-driven process, such that the interaction between D63 from L1 and N260 from L2 with the protonated amine group of GlcN (Fig. 5) brings L1 and L2 close to each other, thus forming the closed roof at subsite (−2) (Fig. 6B).

To further understand how the change in enzyme conformation affects its affinity towards the substrate, ensemble docking was carried out. The binding affinity for the five different conformations of DDDDDD observed in the five separately calculated average enzyme-substrate structures was measured by re-docking each conformation in its corresponding enzyme structure. In the next step, mono-acetylated substrates (ADDDD, DADDDD, DDADDD, DDDADD, DDDDAD, and DDDDDA) generated from each of the five DDDDDD conformations were docked into their corresponding average structure, and the binding scores were measured (Fig. S7). Eventually, the final docking score for the docked substrate was assessed by merging docking scores and calculating the average binding energy for each substrate (Fig. 6D). In this way, we included in the final binding score the effect of the conformational rearrangements that occurred both in the enzyme and in the substrate. Results show that the binding affinity was highest for the completely deacetylated substrate (Fig. 6D), and a GlcNAc residue decreased the binding score at all subsites. Based on docking scores, it is predicted that CSN-MN strongly disfavors GlcNAc at the (−1) subsite; this coincides with our mass spectrometry data (Fig. 3) which show that the enzyme specifically accepts only GlcN at the (−1) subsite. The reason identified for this specificity at subsite (−1) is that residues D135 and S198 (Fig. 5) form stable H-bond interactions with the protonated GlcN subunit, whereas the nearby R274 helps stabilize the charge. Consequently, together the residues D135, S198, and R274 form a compact surface at the inner side of the binding cavity (Fig. S8), thus only providing enough space to accommodate GlcN.

Similarly, in ensemble docking studies for the structures generated from 20–40 ns and 40–60 ns, where the loops formed a closed surface at subsite (−2) (Fig. 6A), steric hindrance caused by the closed cleft prevented DADDDD from fitting into the active site cavity; thus, no docking score was measured (Fig. 6D) at those time points. Similarly, at the same time points, with DDDADD as substrate, negligible or no docking scores were obtained which indicates that the loop movement leading to a closed surface at subsite (−2) also influences subsite (+1) such that it prevents accommodating the acetyl group. Subsite (−3), due to the stable hydrogen bond between the protonated GlcN and D131, has a high preference for GlcN but, as in all enzyme conformations (ensemble structures), ADDDDD was able to fit in the cavity and docking scores were measured which indicates that this site is not completely unavailable to GlcNAc. For subsite (+2), the calculated average binding score for DDDDAD was higher than for the other mono-acetylated substrates, thus suggesting that because of its open site and absence of stable H-bonds, it can generally accommodate both GlcN and GlcNAc, though GlcN is preferred because of the interaction with E263 (Fig. 5). Similarly for subsite (+3), the binding score calculated for DDDDDA was close to that for DDDDAD, indicating that the acetyl group of GlcNAc did not hinder its acceptance at this subsite. Though the acidic residues (D315, E362) present at subsite (+3) might be responsible for giving preference to GlcN, absence of stable hydrogen bond interactions with corresponding residues indicates that this subsite is flexible and, thus, able to accommodate the acetyl group of GlcNAc. Consequently, based on our results, we conclude that GlcNAc is not preferred at subsites (−1), (−2), and (+1), is tolerated slightly more at subsite (−3), and is tolerated the most (but still not preferred) at subsites (+2) and (+3). These ensemble docking results are consistent with our experimental results (Table 1), thus they allow an explanation as to why products carrying a GlcNAc unit at or next to their non-reducing end accumulated after long incubation times (Fig. 3). This accumulation is likely caused by the enzyme’s strong preference for a GlcN unit at subsites (−2) and (−1); as a consequence, products with a GlcNAc unit at the non-reducing end or next to it cannot easily be cleaved further. Indeed, based on our results, we strongly suggest that loop flexibility plays an important role in defining the subsite vs. subunit specificity at subsite (−2).

**Figure 7:**
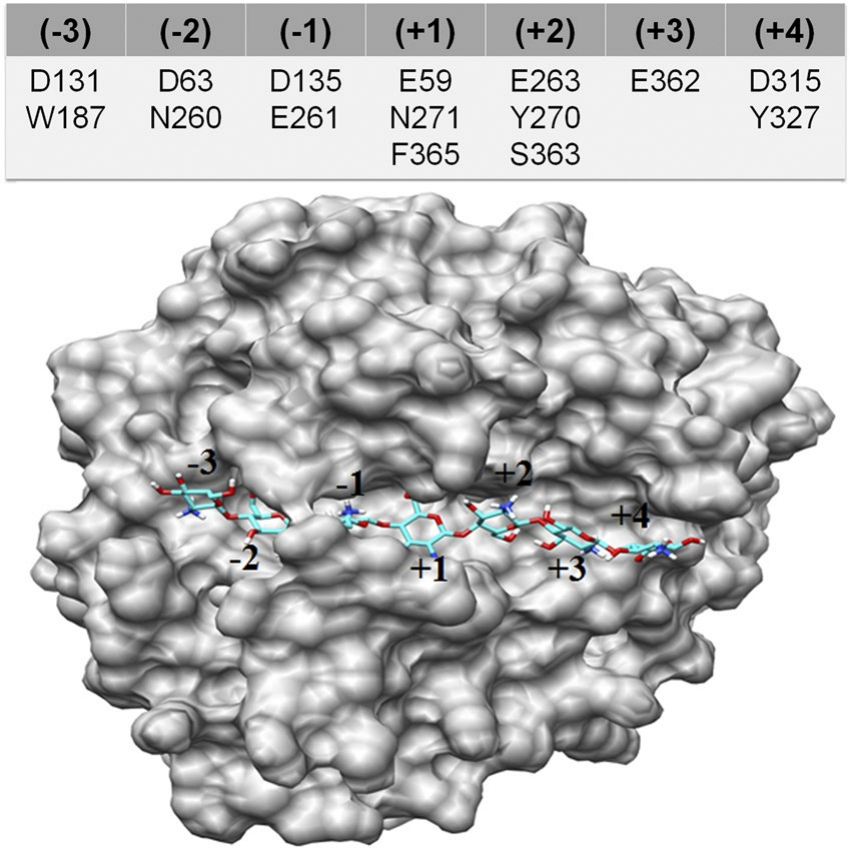
Extra subsite for binding GlcN subunit. Docking of GlcN heptamer on the average CSN-MN structure generated from simulation, displaying the subsite (+4), and the corresponding residues present at each subsite.

Remarkably, during the simulation, the formation of an additional subsite (+4) was observed. To confirm this, using clustering algorithms, we first generated the average structure from the trajectory and proceeded with the docking of the GlcN heptamer as a substrate. As shown in Fig. 7 (PDB: S11), the heptamer fits very well to the generated average structure, where the seventh subunit of the substrate is held by residue Y327 and also possibly by D315 of subsite (+4). Hence, this result clearly indicates that CSN-MN contains seven subsites ranging from (−3) to (+4). Previous studies carried out by Choi *et al*.^35^ on the GH8 chitosanase from *Bacillus sp*. KCTC 0377BP already suggested that the enzyme may contain an extra binding site beyond the six known ones, confirming our results, and vice versa.

### PCA analysis

**Figure 8:**
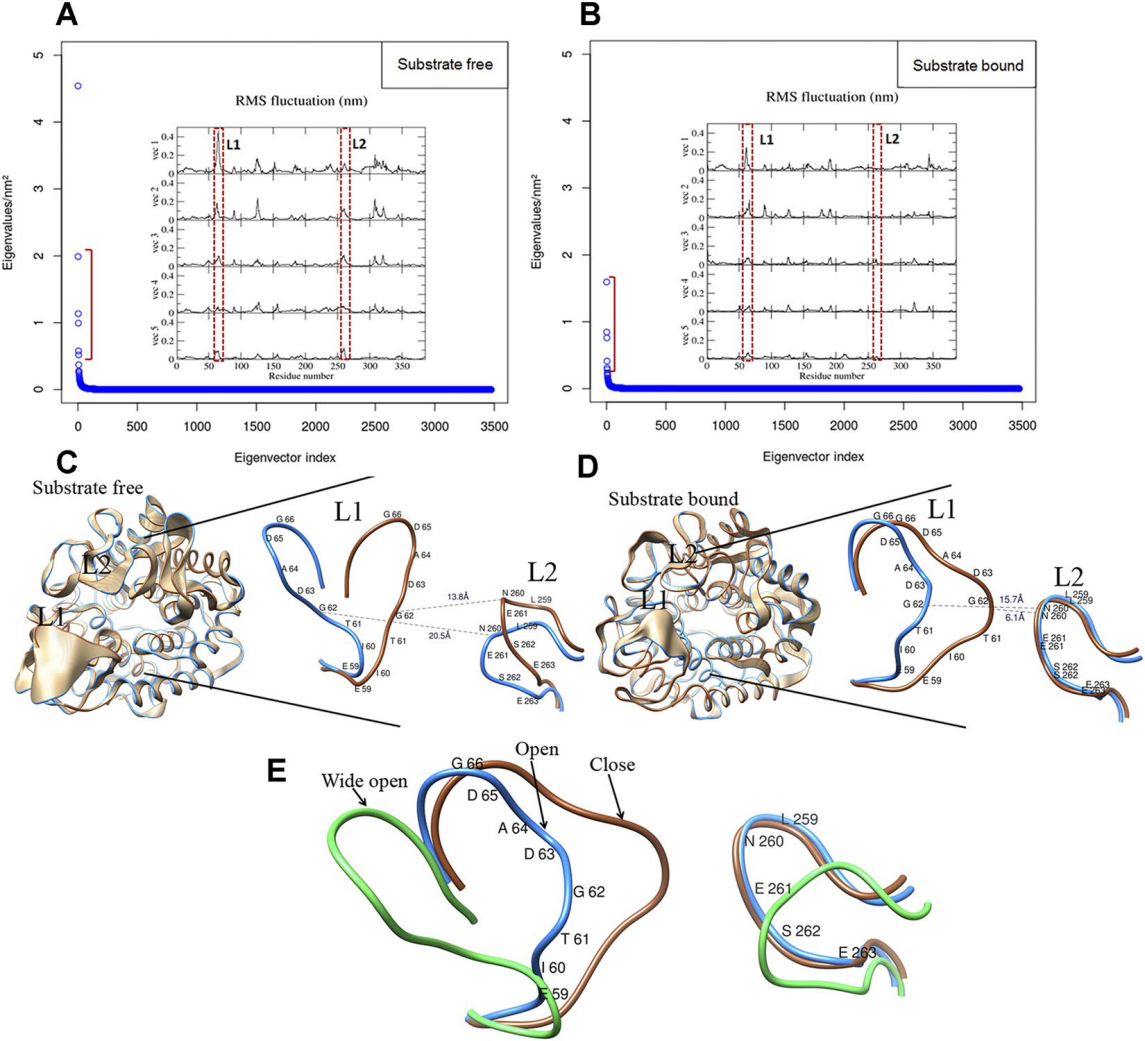
PCA analysis describing the states of the loops derived from substrate-free and -bound enzyme. (**A**,**B**) Principle component analysis: eigenvector and eigenvalues of the covariance matrix in the substrate-free and -bound forms of CSN-MN indicate that motions were higher in the substrate-free enzyme than in the substrate-bound enzyme; graphs in the inset display the per residue fluctuations derived from the protein backbone. (**C**) Extreme structures displaying the cleft opening, calculated from eigenvector 1 from the substrate-free CSN-MN, with the maximum (20.5 Å) and minimum (13.8 Å) distance between loops L1 and L2. (**D**) Extreme structures displaying the cleft opening, calculated from eigenvector 1 from the substrate-bound form of CSN-MN, with the maximum (15.7 Å) and minimum (6.1 Å) distance between loops L1 and L2. E) Based on the loop motion in the substrate-free and -bound enzyme, three states of loop L1 are defined in CSN-MN, i.e. wide open, open, and closed.

To statistically identify the significant collective mode of atomic motions at the binding site in substrate-free and -bound CSN-MN, we performed a principal component analysis (PCA)^36^ on the MD trajectory. Our PCA result shows that in the substrate-free and -bound forms of CSN-MN, the overall dynamic can be described by the first five eigenvectors (Fig. 8A,B). To map the motion onto the structure, we derived root mean square fluctuations (RMSF) from the protein backbone by considering the first five eigenvectors. Amino acid residues mapped from loops L1 and L2 on RMSF data showed that loop L1 fluctuated in both the substrate-free and -bound forms of CSN-MN, while loop L2 slightly fluctuated in the substrate-free form but was completely stabilized in the substrate-bound form. This behavior was relatively similar in all five eigenvectors (RMSF) derived from both forms of the enzyme (Fig. 8A,B), which indicates that the fluctuation in loop L1 is mainly responsible for forming the closed surface at the (−2) subsite upon substrate binding. All the same, stabilization of loop L2 in the substrate-bound enzyme seems to be important for catalytic activity. In the free enzyme, the catalytic residue E261 in loop L2 protruding into the cavity remains positioned between subsites (−2) and (−1), whereas in the substrate-bound form at the very beginning of the simulation, the conformation of L2 changes and E261 moves to a position between subsite (−1) and the catalytic site situated between subsites (−1) and (+1). Apparently, the position of loop L2 is stabilized at the catalytic site via the H-bond interaction between E261 and N271 positioned between the catalytic site and subsite (+1) (Fig. S9B). During the process, the adjacent residue N260 simultaneously moves downward and becomes positioned at the (−2) subsite, where its side chain is involved in the H-bond interaction with the substrate, thus contributing to subsite affinity (Fig. 5). As L2 contributes to both the hydrolysis reaction and substrate affinity, we conclude that the stable conformation of this loop in the enzyme’s substrate-bound form is important for the reaction.

Further, in the substrate-free enzyme, from the extreme structure (representing the maximum projection from the average structure) generated from eigenvector-1, the maximum and minimum amplitude of the opening of the cleft measured from the Cα of G62 in loop L1 to the Cα of N260 in loop L2 were 20.5 Å and 13.8 Å, respectively (Fig. 8C). On the other hand, in the substrate-bound structure, the maximum and minimum extents of cleft opening measured between the residues mentioned above were 15.7 Å and 6.1 Å (Fig. 8D), respectively. Consequently, based on loop distances, we define three states of the loop-delineated active site of the enzyme, i.e. wide-open, open, and closed (Fig. 8E). We suggest that the closed state most likely represents the conformation during catalysis, the open state corresponds to substrate binding and product release in the high affinity state (due to loop interaction with the substrate), while the wide-open one is related to substrate binding in the low affinity state. To visualize the loop movements at subsite (−2), we generated a movie from the first eigenvector (Movie S12). These results clearly explain the enzyme kinetics based on an induced fit mechanism in chitosanase. This phenomenon of open and closed states at subsite (−2) of the active site has been observed before only for a GH46 chitosanase (CSN OU01)^23^. A related GH46 chitosanase which shares 58% sequence identity with CSN OU01, namely CSN-174, was recently shown to share specificity for GlcN at subsite (−2)^17^ with the here described GH8 chitosanase CSN-MN, indicating that (−2) subsite specificity might be conserved in different families of chitosanases due to the involvement of loops in opening and closing the cleft, and their interactions with the substrate. Thus, we suggest that probably the closed surface at the binding site created by the movement of loops is required for efficient hydrolysis by chitosanases of families GH8 and GH46. Both, though not related in amino acid sequence, nonetheless share a similar mode of action.

## Conclusions

Chitosans are promising functional biopolymers with various applications. The biological activities of partially acetylated chitosans often depend on the functionalities of oligomers enzymatically produced from them. Thus, well-defined chitosan oligomers are required to investigate the biological activity of chitosans. Chitosanolytic enzymes have been proposed as tools to produce chitosan oligomers of at least partially known and defined structure in terms of DA, DP, and PA. Hence, determining chitosan degrading enzymes’ subsite specificities and preferences is very useful for producing chitosan oligomers with more defined structure. To our knowledge, this study is the first to present a detailed picture of a GH8 chitosanase concerning its mode of action and subsite specificity. This knowledge can guide the design of muteins with modified subsite specificities by targeting the residues responsible for the specificity of a subsite of interest. Targeted modification of subsite specificities will yield oligomeric products with targeted, modified patterns of acetylation. This will allow generating chitosan oligomers with different, defined architectures than currently available, and those can then be tested for their suitability in various biotechnological applications. This approach has already been successfully applied to loosen, by site-directed mutagenesis, the tight specificity of CSN-MN for GlcN at subsite (−2), creating muteins that readily accept GlcNAc at this site and, consequently, produce chitosan oligomers with patterns of acetylation that differ from the products of the wild type enzyme^20^.

## Materials and Methods

### Bacterial strain, culture conditions, and enzyme purification

CSN-MN was prepared using the strain *E*. *coli* Rosetta2 (DE3) [pLysSRARE2; pET22b::StrepII-CSN]. Culture conditions and enzyme purification were performed as described in Nampally *et al*.^19^.

### Oligomeric and polymeric chitosan substrates

Chitosans with different DAs were prepared via partial re-*N*-acetylation^37^ of a chitosan with DA 1.6% and DP 1400, kindly provided by Mahtani Chitosan PVT. LTD. (Veraval, India). The resulting DAs were validated by ^1^H-NMR^38^. GlcN_1–6_ oligomers were purchased from Carbosynth Limited (Compton, UK).

### Thin layer chromatography (TLC) of oligomeric products

Fully de-*N*-acetylated chitosan oligomers of different DP (dimers to hexamers, GlcN_2_ - GlcN_6_, 0.5 mg/ml, 10 µl) were incubated for 1 h at 37 °C in 25 mM ammonium acetate buffer (pH 5.2) containing 0.2 µM CSN-MN. Products were separated using silica gel HP-TLC (CAMAG automatic TLC sampler) using a 5:4:2:1 (v/v/v/v) mixture of *n*-butanol, methanol, 25% ammonia, and water. Chitosan oligomers were detected by spraying the plate with 30% (w/v) ammonium sulfate and heating it using a hot air gun until dark bands appeared. From chromatography plates, images were acquired with Adobe Photoshop, brightness and contrast of scanned images was adjusted with MS-office tool.

### Enzymatic hydrolysis of fully De-*N*-acetylated chitosan polymer

Chitosan polymer (DA 0%, 1 mg/ml) was incubated at 37 °C for 1, 2, 3, 4, 5, 15, 30, and 60 min in 50 mM ammonium acetate buffer (pH 5.2) containing 25 nM CSN-MN. Aliquots of each sample (20 µl) were inactivated by adding 1 volume of NH_4_OH (10%) and heating at 95 °C for 2.5 min. Reaction products were analyzed using TLC as described above.

### Enzymatic hydrolysis of partially acetylated chitosan polymers

For hydrolysis, chitosan polymers (DA 2–50%, 1 mg/ml, 100 µl) were incubated at 37 °C for 42 h in 40 mM ammonium acetate buffer (pH 5.2) containing 0.2 µM CSN-MN. Reactions were stopped by heating at 95 °C for 2.5 min and the hydrolysis products were analyzed using HP-TLC as described above.

### Reducing end assay

The amounts of reducing ends produced by enzymatic hydrolysis were determined according to Horn and Eijsink^39^. GlcN in the range of 0.05–1.75 µM was used as the standard. To test the substrate specificity, 0.5 mg/ml of chitosans of DA 2–48% were incubated under mild shaking at 37 °C for 48 h in 40 mM sodium acetate buffer (pH 6) containing 0.1 µM CSN-MN. Enzyme-free samples were used as blanks for each substrate. For kinetics, different concentrations of chitosan DA 10% were incubated for 4 min at 50 °C under mild shaking in 50 mM sodium acetate buffer (pH 6) containing 0.03 µM CSN-MN. To calculate the initial enzyme activity, an additional sample was taken from each reaction mixture after 15 sec. All samples were inactivated with 1 volume of 0.5 M NaOH. Initial reaction velocities were plotted against the substrate concentration and fitted with a three parameter Hill fit using the software Sigma Plot 12.5 (Systat Software GmbH, Erkrath, Germany).

### MALDI-TOF MS analysis of chitosan oligomers

MALDI-TOF mass spectrometric (MS) analysis was carried out on enzymatically degraded chitosan oligomers, using a 10 mg/ml aqueous solution of 2,5-dihydroxybenzoic acid (DHB) as a matrix. One µl of the matrix was mixed with 1 µl of sample (1 mg/ml). MALDI mass spectra were recorded on Autoflex Speed (Bruker Daltonics, Bremen, Germany). For acquiring the spectra, a SmartBeam^TM^ NdYAG-Laser with a wavelength of 355 nm was used. Mass spectra were analyzed with mMass (mmass.org/features/peaklist.php).

### ESI MS analysis of chitosan oligomers

MS^2^ analyses of mono-deacetylated CSN-MN products (i.e. GlcN_n_GlcNAc, with n ≥ 1) were carried out by dissolving 5 µg of chitosan oligomers in 10 µl of ^18^O-labelled water and 0.1% formic acid. Samples were incubated overnight at 70 °C to exchange the hydroxyl group at the reducing end of oligomers with ^18^OH. Samples were freeze-dried and dissolved in 10 µl deionized water, and 1 µl of this solution was used for LC-MS using a Waters Acquity BEH Amide column (1.7 µm, 2.1 mm × 150 mm) in a Dionex Ultimate 3000 UHPLC coupled to an ESI-mass spectrometer (Amazon speed, Bruker Daltonics). MS^2^ was performed in positive mode and data were evaluated using the Bruker Compass Data Analysis program. The abundance of different mono- deacetylated chitosan oligomers was quantified based on the method described by Cord-Landwehr *et al*.^40^. Briefly, oligomers were re-*N*-acetylated using [^2^H_6_] acetic anhydride and labelled at the reducing ends using H_2_^18^O. To determine the abundance of different oligomer sequences, the largest b-ion and all y-ions of each precursor were considered. Scripts written in the Python programming language were used to evaluate the sequencing data^41^.

### Molecular modelling

A 3D model of CSN-MN was generated using the MODELLER package^42^. The crystal structure of chitosanase from *Bacillus sp*. K17^24^ (PDB: 1V5C) served as a template (96% identity). The KoBaMIN^43^ server was applied for post-refinement and stereo-chemical correction. Geometric accuracy of the refined model was evaluated using MOLPROBITY^44^. UCSF CHIMERA^45^ was used for calculating RMSD. N-acetyl glucosamine oligomers were retrieved using POLYS database (http://glycam.org), and glucosamine subunits were modeled using the molecular modeling program UCSF CHIMERA^45^ and AVOGADRO^46^. Modeled oligomers were energy minimized by implementing the MMFF94^47^ force field.

### Electrostatic charge distribution and docking studies

The adaptive Poisson-Boltzmann Solver (APBS)^48,49^ program was used to calculate the electrostatic distribution on the CSN-MN model, and PYMOL^50^ was applied to visualize the surface representation. For docking studies, AUTODOCK v4.2^51^ was used. The substrate and protein structures were prepared for docking simulation by adding hydrogens, merging nonpolar hydrogens, and assigning charges. Docking grid size was prepared with the *autogrid* utility in AUTODOCK; the grid center was placed in the middle of the active site and was adjusted so that the grid boxes included the entire active site. The *Lamarckian Genetic Algorithm* (LGA) was use to search for the best conformations. The binding energy between substrate and enzyme was evaluated using *autoscorer*. Initially, for generating enzyme-substrate complexes, docking was carried out by giving maximum flexibility to the substrate. Further, for ensemble docking, 100 ns (Fig. S7) simulation trajectories were divided into five parts and average enzyme-substrate conformations were calculated from each part by implementing a clustering algorithm. From each enzyme-substrate conformation, GlcN_6_ conformation was removed and re-docked by restricting the torsion angle of substrate glycosidic bonds, and the binding affinity was measured for each conformation. Subsequently, mono-acetylated oligomers were generated by placing the acetyl group in each GlcN_6_ conformation and rigid docking was carried out with mono-acetylated oligomers to score the effect of the acetyl group.

### Molecular dynamics simulations

Three independent molecular dynamics (MD) simulations of 100 ns each were carried out on substrate-free and substrate-bound CSN-MN. All simulations were performed using GROMACS 5.0.2^52^ software. The Gromos96–53a6 force field was used on proteins with and without a bound substrate. GlcN_6_ topology was generated using PRODRG server^53^. Simulations were run with protonated catalytic residue E74, in both bound and unbound cases, the protein was individually solvated in the cubic water box of the SPCE water model with box edges of 1 Å from the protein periphery. To provide a neutral system, nine Na^+^ counter ions were added. The system was pre-equilibrated by minimization using the steepest descent method, followed by second energy minimization at a constant volume and temperature (300 K) for 500 ps, while restraining the atomic positions of the protein. Simulations were run with periodic boundary condition (PBC). Leap-frog algorithm^54^ was applied for the integration of Newton’s equations of motion with a time step of 2 fs and particle mesh Ewald (PME) algorithm^55^ was used for long-range electrostatics interactions. The final production MD was run for 100 ns. Snapshots were collected every 2 ps. GROMACS inbuilt utilities were used for calculating RMSD, radius of gyration, potential energies, and dihedral angles. Hydrogen bonds between the amino acid residues and the protonated glucosamine subunits were calculated on the basis of donor-acceptor distances smaller than 3.5 Å. Protein substrate structures were clustered using the clustering tool available in GROMACS with cutoff 1 Å. Principal component analysis (PCA) was performed on the substrate-free and -bound forms of CSN-MN. In the first step of PCA, a covariance matrix corresponding to the main chain of the enzyme was constructed, capturing the degree of co-linearity of atomic motions for each pair of atoms. By diagonalization of the covariance matrix, eigenvalues and eigenvectors were obtained. The eigenvector describes the collective motion of atoms, and eigenvalues represent the degree to which the corresponding atom participates in the motion. GROMACS inbuilt utilities were used for generating the covariance matrix and for analyzing eigenvectors from backbone atoms.

## Electronic supplementary material

Supplementary

Movie S12. Loop movement at subsite (-2)

Supplementary material 1

Supplementary material 2
