# Supplementary material for: Structural and biochemical insight into mode of action and subsite specificity of a chitosan degrading enzyme from Bacillus spec. MN
Source: Sci Rep. 2019 Feb 4;9:1132. doi: 10.1038/s41598-018-36213-6 (PMC6362164; doi:10.1038/s41598-018-36213-6)
Supplement: Supplementary file 1 — Supplementary [file 41598_2018_36213_MOESM1_ESM.pdf]

Supplementary Information

Structural and biochemical insight into mode of action and subsite specificity of a chitosan degrading enzyme from *Bacillus spec.* MN

Ratna Singh, Tobias Weikert, Sven Basa, Bruno M. Moerschbacher*

Institute for Biology and Biotechnology of Plants

University of Münster, Schlossplatz 8, 48143 Münster, Germany

Supplemental figures with legends:

Figure S1. Chitosan oligomer and polymer chromatography

Figure S2. Semi-native PAGE of CSN-MN

Figure S3. MALDI-TOF MS spectra

Figure S4. Reducing end assay

Figure S5. Superimposed structure of CSN-MN with docked $GlcN_6$ and CelA bound with cellopentaose

Figure S6. Assessment of stability of trajectory

Figure S7. Ensemble docking protocol

Figure S8. Subsite specificity at (-1) and (-2) subsite

Figure S9. Residues stabilizing the L2 loop at the catalytic site

PDB S10. Substrate bound CSN-MN 3D structure

PDB S11. Heptamer bound CSN-MN 3D structure

Movie S12. Loop movement at subsite (-2)

Supplemental Figures

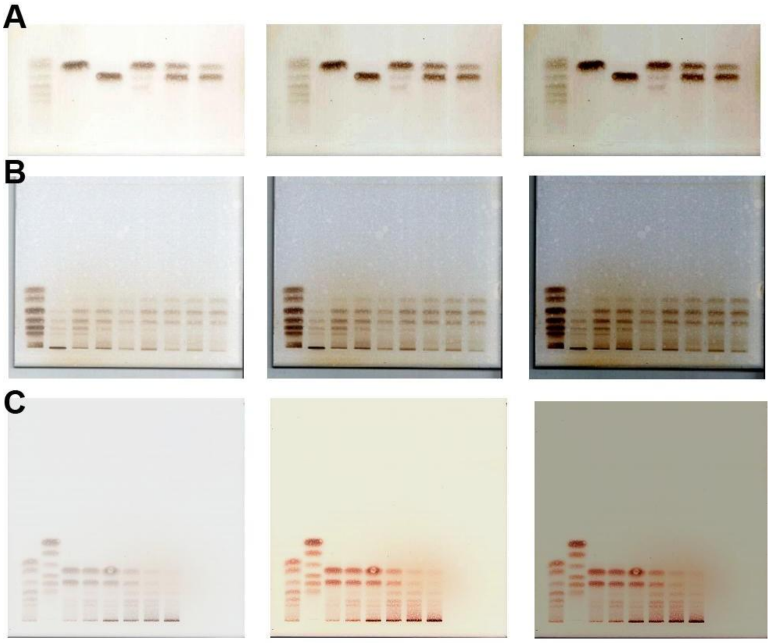

Figure S1 - Uncropped images at different levels of contrast and brightness from thin layer chromatography of chitosan oligomer and polymer degraded with CSN-MN

(A) Thin layer chromatography of chitosan oligomers obtained by digestion of GlcN (D) oligomers. (B) Degradation of chitosan polymers of DA 0% at different time (C) A range of chitosan polymers with varying DA from 2 to 50%. D, GlcN; A, GlcNAc.

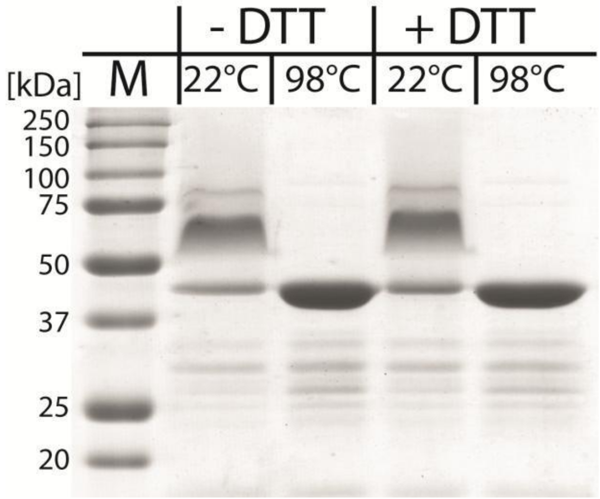

Figure S2. Semi-native PAGE of *Bsp*CsnMN

Separation was achieved on a 12% polyacrylamide gel. Samples were incubated for 2 min at 22°C or 98°C in the presence or absence of 25 mM DTT. Precision Plus Protein™ All Blue Prestained Protein Standard (Bio-Rad Laboratories GmbH, Munich, Germany).

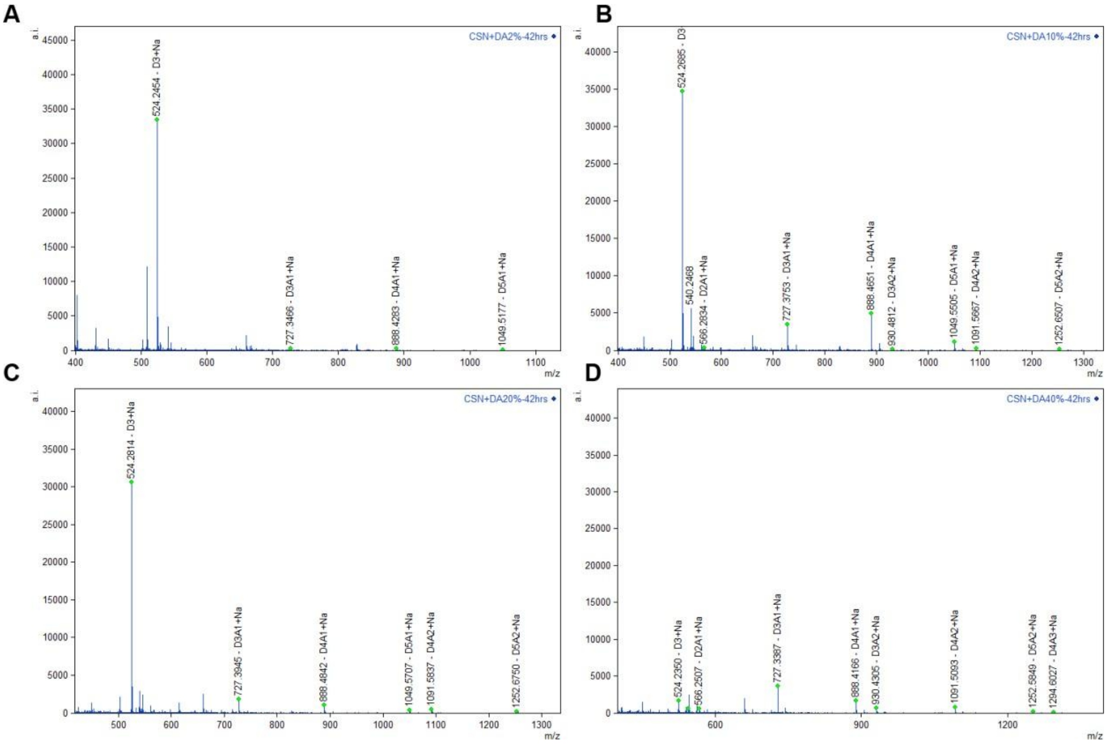

Figure S3. MALDI-TOF MS spectra

Mass spectra of oligomeric products obtained by CSN-MN catalyzed hydrolysis of chitosan polymers (A) degree of acetylation of 2% (B) degree of acetylation of 10% (C) degree of acetylation of 20%, and (D) degree of acetylation of 40%; D, GlcN; A, GlcNAc.

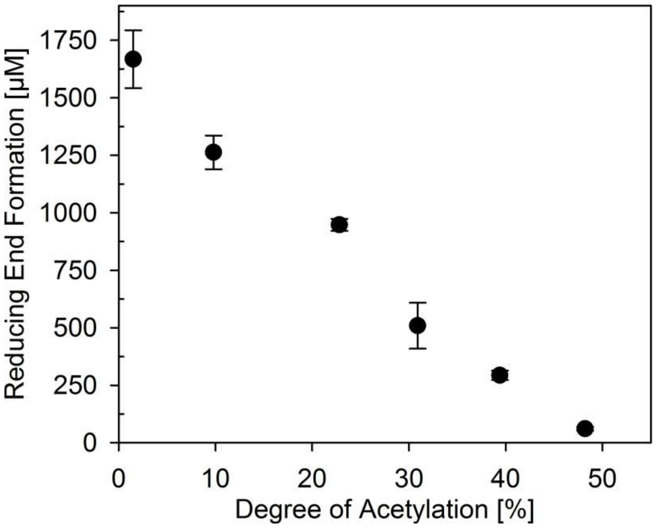

Figure S4. Reducing end assay

Reducing ends formed by CSN-MN digestion of chitosan polymers with different degrees of acetylation.

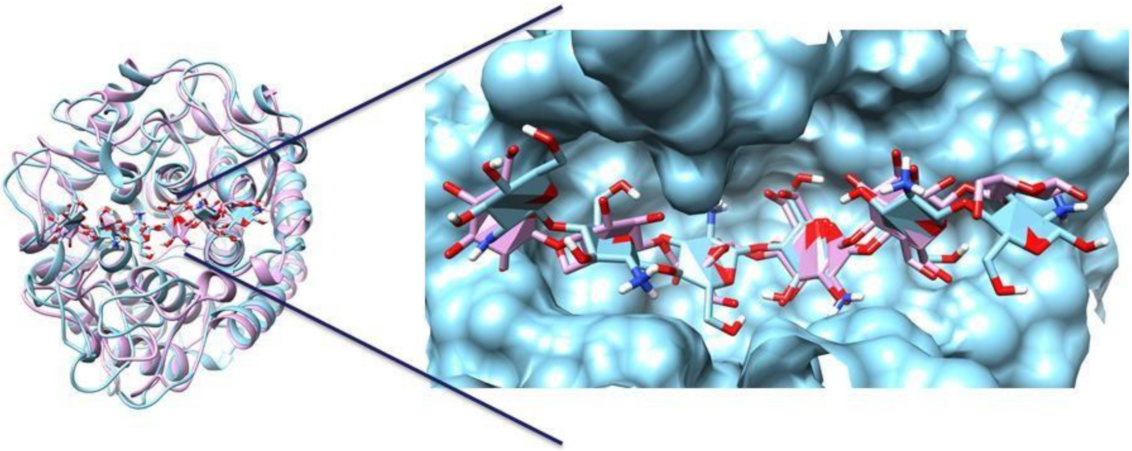

Figure S5. Superimposed structure of CSN-MN with docked $GlcN_6$ and CelA bound with cellopentaose

CSN-MN structure with docked $GlcN_6$ substrate (blue color) superimposed on the endoglucanase CelA structure (1KWF.pdb) bound with cellopentose (purple color), displaying the similarity of the substrate conformation at the binding site and the accuracy of the docking procedure.

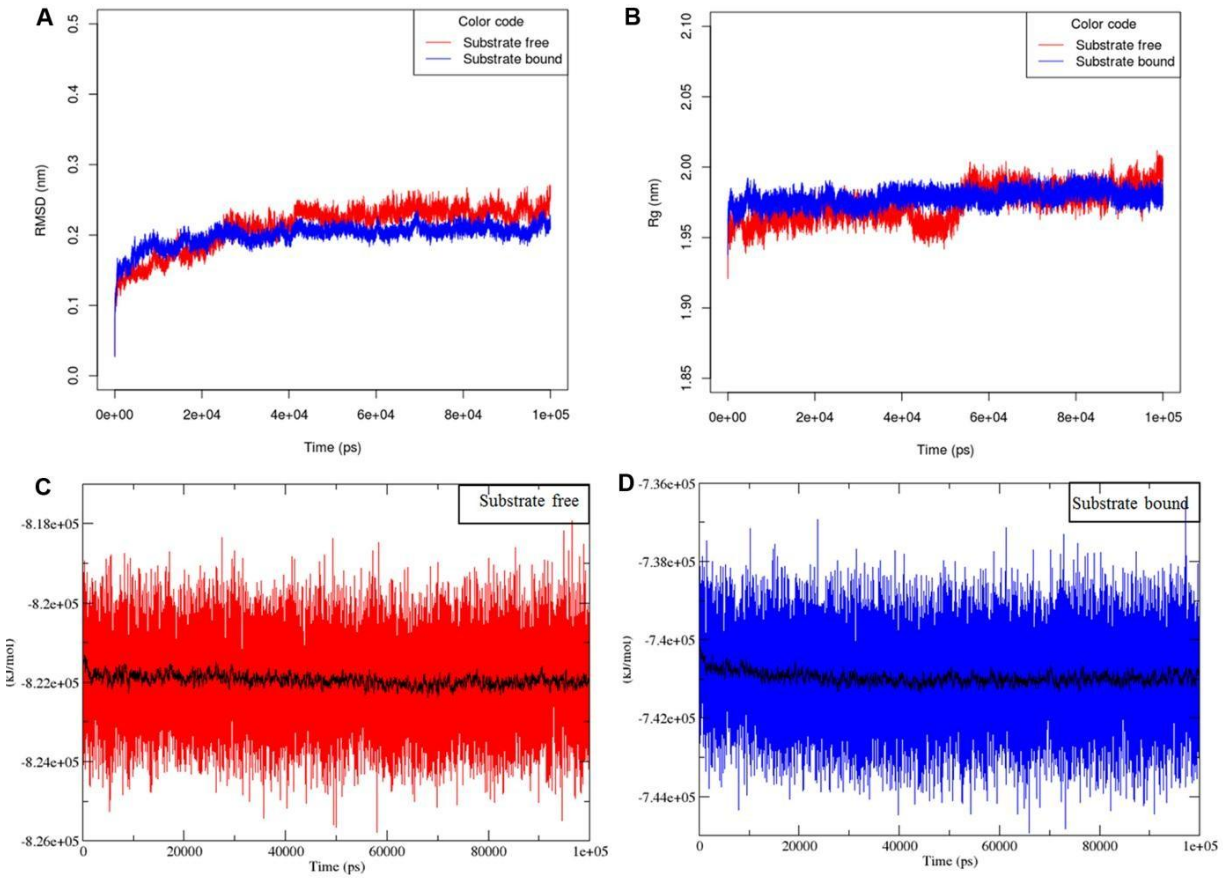

Figure S6. Assessment of stability of trajectory

(A) Time dependence root mean square deviation (RMSD) in the substrate free and substrate bound CSN-MN. Overall RMSD measured from Cα remained below 2 Å throughout the simulation. (B) Radius of gyration (Rg) indicates the compactness of protein structure. The mean Rg calculated for substrate free enzyme was 1.99 ± 0.010 nm and substrate bound enzyme was 1.97 ± 0.005 nm, indicating the stability of structure. (C) Potential energy during simulation in substrate free CSN-MN, black line representing average value after every 100 steps indicating stable simulation. (D) Potential energy during simulation in substrate bound CSN-MN, black line representing average value after every 100 steps indicating stable simulation.

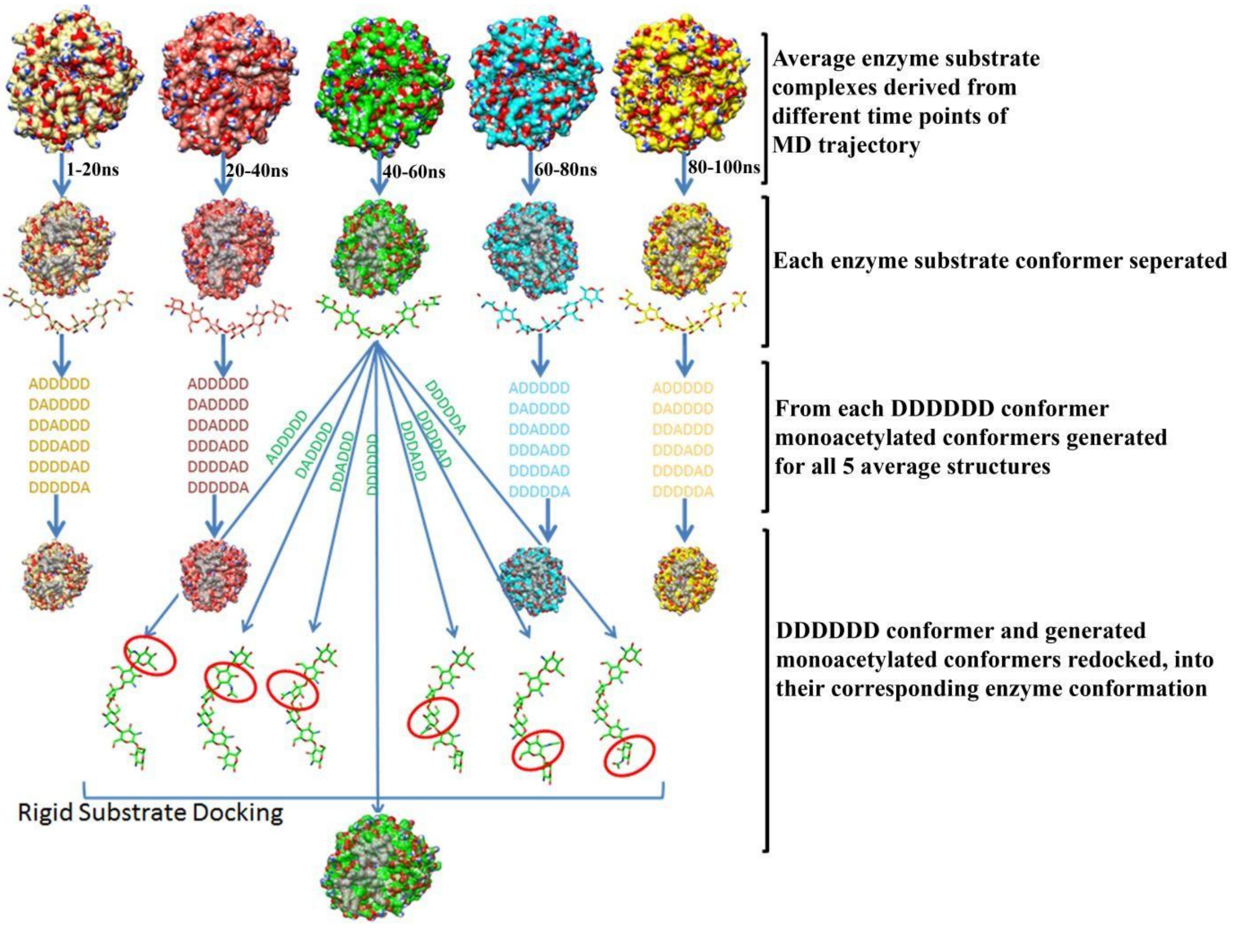

Figure S7. **Ensemble docking protocol**

Molecular dynamic simulation combines with docking and each conformer was scored. Average score was calculated for each monoacetylated substrate as final binding score.

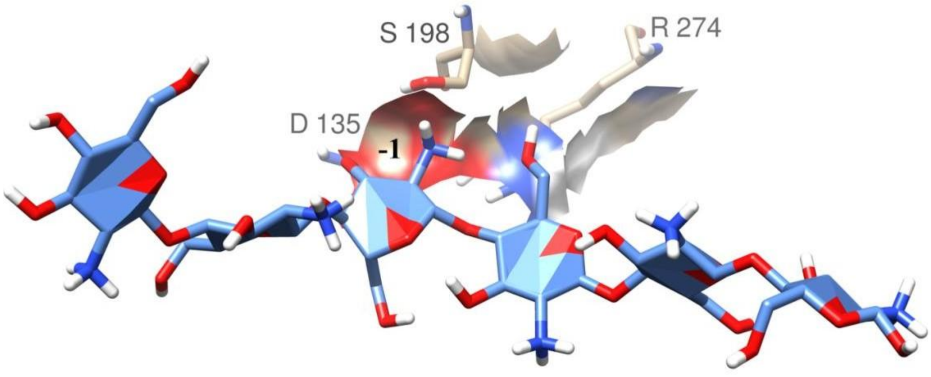

Figure S8. Subsite specificity at subsite (-1)

Amino acid residues D135, S198, R274 form a compact cavity around amine group of GlcN at (-1) subsite providing reason for GlcN specificity.

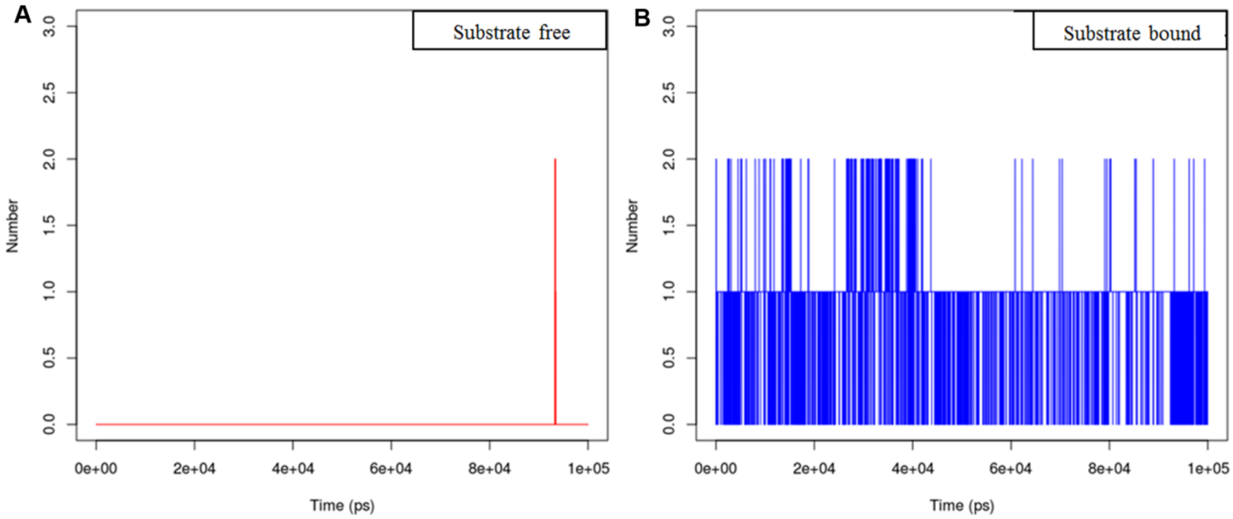

Figure S9. **Residues stabilizing the L2 loop at the catalytic site**

(A) H-bond interaction between E261 and N271 in substrate-free enzyme, displaying almost no interaction between the residues. (B) H-bond interaction between E261 and N271 in substrate-bound enzyme indicates that N271 contributes in holding residue E261 at catalytic site.
